# Comparative Evaluation of Effects of Oral Diltiazem and Topical Diltiazem (2%) Ointment in the Treatment of Chronic Anal Fissure: A Prospective Randomized Study

**DOI:** 10.34172/mejdd.2024.383

**Published:** 2024-07-31

**Authors:** Ekta Sharma, Pankaj Dugg, Nisha Rani, Vivek Pahuja, Sushil Kumar Mittal, Harnam Singh Rekhi

**Affiliations:** ^1^Department of General Surgery, Government Medical College and Hospital, Sec-32, Chandigarh, India; ^2^Department of General Surgery, Government Medical College, Amritsar, India; ^3^Civil Hospital, SAS Nagar, Punjab, India; ^4^Department of General Surgery, Adesh Medical College and Hospital, Vill Mohri, Tehsil- Shahbad, India

**Keywords:** Anal fissure, Diltiazem, Pain, Bleeding

## Abstract

**Background:**

Fissure-in-Ano is a common condition of the anorectal region. Most of the time, it is managed non-surgically. There are various drugs used for the treatment of anal fissures. Calcium channel blockers are one of them that reduce the tone of sphincter muscles. The present study compares the efficacy of oral diltiazem and topical 2% diltiazem ointment in patients with chronic anal fissures.

**Methods:**

Patients were randomized into two groups. Group A (n=25) received treatment in the form of oral diltiazem, while group B (n=25) received treatment in the form of 2% (weight/volume) diltiazem ointment for local application in addition to other conservative methods like sitz bath and stool softeners. Outcomes in the form of success of treatment and complications were assessed. Statistical analysis was done using MedCalc software version 14.0. *P* value of<0.05 was considered significant.

**Results:**

The mean age of patients was 32.00±10.67 years in group A and 30.64±9.53 years in group B. Pain relief was significantly better in group B than in group A at the end of the first week (*P*=0.00018), but at the end of 6th week, no significant difference was observed. Fissure healing was more significant in group B than in group A after 6 weeks (*P*=0.0152).

**Conclusion:**

Local diltiazem ointment is a better option than oral diltiazem for anal fissures with respect to better outcomes and lesser complications.

## Introduction

 Fissure -in- ano/anal fissure is a common condition of the anorectal region that usually presents as anal pain while defecating.^1^ The fissures can be acute (lasting for < 6 weeks) or chronic (lasting for > 6 weeks). The incidence of anal fissures is approximately 1 in 350 adults. They are commonly seen equally in men and women of the adult age group (15-40 years).^2^

 The most frequent cause, probably, and the initiating factor of fissure-in-ano is trauma to the anal canal while passing hard stools.^1^ In women, it is usually triggered during pregnancy and following childbirth due to changes in dietary habits. Irritable pre-existing anal canal conditions have also been postulated, leading to fissures.^3^ Scarring, stricture, and stenosis because of anal injury due to any previous surgical procedure are recognized conditions that predispose to fissure formation.^1^

 High resting anal pressure, which is because of increased activity of the internal anal sphincter, is seen in almost all patients (except in postpartum patients) with chronic anal fissures. Ambulatory manometry has confirmed the presence of sustained resting hypertonia in patients with fissures.^4^

 A tearing or glass-cutting pain while defecating is by far the most common symptom of anal fissure. The pain because of anal fissure lasts for minutes to hours, and in patients with a chronic fissure, it is most commonly described as profound anal “tightness” or “spasm’’. Exposure of internal anal sphincter muscle underneath the fissure results in a spasm of muscle, causing severe pain.^5^

 It is estimated that half of all the patients will heal with non-operative measures like a warm sitz bath, an increase in intake of fiber-rich diets (psyllium fibers), stool softener agents, an increase in intake of liquids, topical application of local anesthetic agents, chemical sphincterotomy. Rest may require surgical intervention.^6^

 A variety of agents like botulinum toxin, glyceryl trinitrate, and calcium channel blockers like nifedipine & oral diltiazem have been used to date for local application. All these agents act by breaking the cycle of pain, spasm, and ischemia. Botulinum toxin was the first agent to be used in 1993.^6^ Diltiazem, a calcium channel blocker, is widely and safely used in clinical practice as an antihypertensive and anti-anginal agent based on the principle that it causes relaxation of vascular smooth muscles and causes vasodilation.^7^ On the basis of the same principle, oral and topical preparations of diltiazem have been used, which decreases the anal resting pressure.^8^

 The present study was conducted to compare and evaluate the efficacy of oral and topical diltiazem in the management of chronic anal fissures.

## Materials and Methods

 The prospective study was conducted on an outpatient basis in the Department of General Surgery at a tertiary care hospital after obtaining permission from the institutional ethics committe (S.No. 38, BFUHS/TH/9972 dated 30/07/2021). A total of 50 patients presenting with chronic anal fissures and fulfilling all selection criteria were included in the present study. Patients were informed about the procedure of the study and its related complications in their own verbal language. Written informed consent was obtained from every patient.

 All patients were divided into two groups using computer-generated randomization.

 Group A included 25 patients who were treated with oral diltiazem (diltiazem tablets 60 mg twice daily) along with other conservative methods like sitz bath and stool softeners. Group B included 25 patients who were managed with topical diltiazem ointment (2% w/v twice daily) along with other conservative methods like sitz bath and stool softeners. The patients in both the groups were supposed to take treatment for a duration of a total 6 weeks.

###  Inclusion Criteria

1. All the cases of single anal fissure, either anterior or posterior, of more than 6 weeks duration.
2. All fissures with associated features of chronicity like sentinel piles or hypertrophied papillae or exposure of horizontal fibers of internal sphincter.

###  Exclusion Criteria

1. Acute anal fissures, either single or multiple, of < 6 weeks duration without any secondary changes.
2. Patients already taking nitrates, calcium channel blockers or beta antagonists for other clinical indications.
3. Fissures in pregnant and breast-feeding women.
4. Women not using reliable contraception.
5. Patients not willing to give written informed consent.
6. Known cases of Crohn’s disease, HIV, tuberculosis, fistula-in-ano or anal cancer presenting with anal fissure.
7. Previous allergic or sensitivity reactions to diltiazem.
8. Patient with a history of chronic headache.

###  Methodology

 After diagnosing patients as having chronic anal fissures, a detailed history and clinical examination were recorded on pretested proforma. The intensity of pain was assessed before and during the course of treatment by using a visual analog scale (VAS). The VAS is a 10 cm line on which 0 represents no pain, and 10 represents the most severe pain.

 Group A patients were prescribed oral diltiazem, while group B patients were treated with topical 2 % diltiazem ointment.

 Patients of group B were instructed to apply the diltiazem ointment locally twice daily, once in the morning after passing stool and then again during the night before going to bed. It was to be applied by fingertip after proper lubrication in the lumen with rotatory movements. The fingertip was supposed to be kept for 20-30 seconds and was gently removed. The amount of ointment recommended for each application was the size of a pea or the tip of the index finger (0.5 gm). The patients were encouraged to follow a high-fiber diet and use a warm sitz bath (before applying the ointment in group B).

 The patients under study were initially followed up twice a week during the first week of follow-up to find the relief of pain, which was assessed and recorded on VAS, and after that, the same procedure was followed on 3 and 6 weeks to assess for fissure healing. During the course of treatment, the time taken for complete healing of the fissure (in the form of resolution of symptoms like anal pain and bleeding and absence of fissure on examination) was noted. Upon complete healing of the fissure, the patients were asked to stop taking medicine and continue a high-fiber diet. The healed fissures were then subsequently followed up to 3 months for recurrence, if any. Also, the side effects of treatment, particularly headache, postural hypotension, palpitation, and dizziness, were noted during each follow-up.

###  Assessment Tools

 The intensity of pain was assessed by using a VAS. For follow-up, all participants were given a questionnaire containing a VAS for pain management. A score of 0 to 2 was graded as mild pain, 3 to 6 as moderate pain, and a score of greater than 6 as severe pain.

 Healing was assessed by resolution of symptoms (anal pain and bleeding) and the absence of a fissure on examination.

 Patients not showing any improvements at 6 weeks were labeled as non-healers or failures of medical treatment and were then offered the opportunity to undergo surgical intervention for the further management of the disease.

###  Statistical Analysis

 At the end of the study, all data were entered into an Excel sheet, and they were analyzed statistically using the MedCalc software version 14.0. In the case of non-parametric distribution of quantitative data, the data were summarised in the form of median [median-range (min.-max.)], and inter-group comparison was made by the Mann-Whitney U test. A *P* value < 0.05 was considered statistically significant.

## Results

 The mean age of group A was 32.00 ± 10.67 with a range between 18 to 65 years, and the mean age for group B was 30.64 ± 9.53 with a range between 20 to 53 years. In both groups, the maximum number of patients belonged to young age groups i.e. 20-29 years. Age distribution and mean age between the two groups were comparable, and there was no significant statistical difference in terms of age distribution (*P* > 0.05) between the two groups, as depicted in Table 1. 16 out of 25 (64%) subjects in group A were men, and the remaining nine (36%) were women. In group B, 15 out of 25 (60%) subjects were men, and the remaining 10 (40%) subjects were women. There was no significant statistical difference in terms of sex distribution between the two groups.

**Table 1:** Demographic profile

**Age group** **(y)**	**Group A**			**Group B**		
	**Men**	**Women**	**Total**	**Men**	**Women**	**Total**
< 20	1	0	1 (4%)	0	0	0 (0%)
20-29	6	4	10 (40%)	9	4	13 (52%)
30-39	5	4	9 (36%)	3	3	6 (24%)
40-49	2	1	3 (12%)	2	3	5 (20%)
50-59	1	0	1 (4%)	1	0	1 (4%)
≥ 60	1	0	1 (4%)	0	0	0 (0%)
Total	16	9	25	15	10	25
Mean ± SD	32.00 ± 10.67			30.64 ± 9.53		

 The symptoms shown by the patients were:

- Bleeding: In group A, 19 out of 25 patients (76%) had bleeding per rectum, whereas in group B, 20 out of 25 patients (80%) had bleeding per rectum.
- Constipation: In group A, 20 out of 25 (80%) had constipation, whereas in group B, 19 out of 25 (76%) had constipation.
- Pruritus-Ani: In group A, 11 out of 25 (44%) had pruritus-ani, whereas in group B, 9 out of 25 (36%) had pruritus -ani.
- Anal tag: In group A, 20 out of 25 (80%) had anal tag, whereas in group B, 19 out of 25(76%) had anal tag.

**Figure 1:**
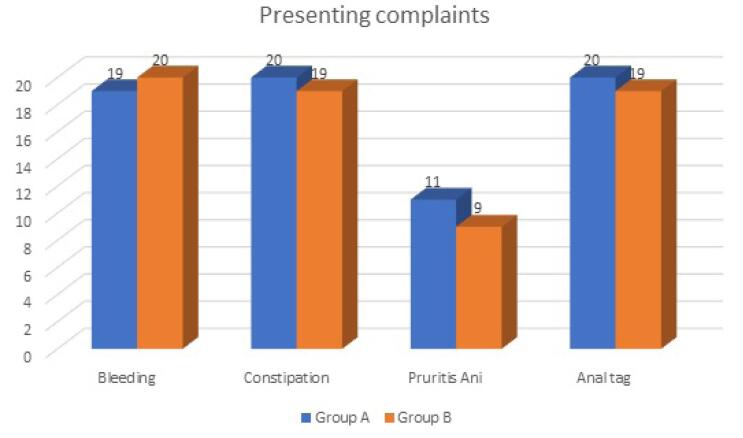
Presenting complaints

There was no significant statistical difference (*P* > 0.05) between the two groups in terms of the presence of symptoms. The findings suggested that most patients in either group had bleeding per rectum along with constipation as part of the disease process (Figure 1).

**Figure 2:**
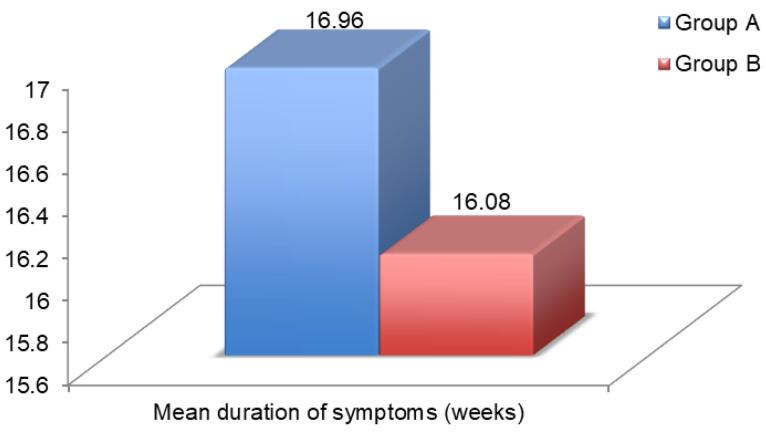
Duration of symptoms

 The mean duration of symptoms in group A was 16.96 ± 12.11 weeks, whereas in group B it was 16.08 ± 11.98 weeks. There was no significant statistical difference in the mean duration of symptoms between the two groups (*P* = 0.756) (Figure 2).

 Patients in both groups complained of pain during defecation along with bleeding per rectum off and on. Pain was assessed using VAS. A score of 0 to 2 was graded as mild pain, 3 to 6 as moderate pain, and a score of more than 6 as severe pain. The median pain score of patients before treatment in group A was 9 (7-10), whereas in group B was 9 (6-10). The two groups were comparable in terms of pain scores before treatment (Table 2).

**Table 2:** Level of pain before treatment

	**Group A**	**Group B**	***P*** ** value**
Median pain score	9 (7-10)	9 (6-10)	0.952

**Figure 3:**
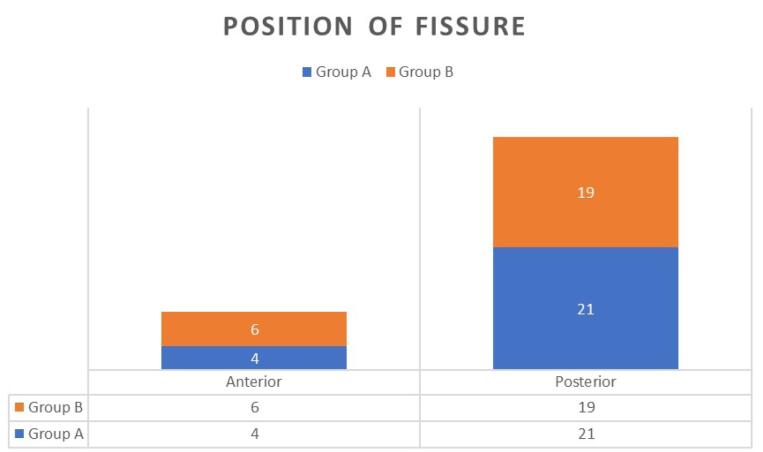
Position of fissure

 Position of fissure in the present study revealed that 21 out of 25 (84%) patients had a fissure in the posterior midline, and 4 out of 25 (16%) patients had a fissure in the anterior midline in group A, whereas in group B, 19 out of 25 (76%) patients had a fissure in the posterior midline and 6 out of 25 (24%) patients had anterior midline fissure. There was no significant statistical difference (*P* = 0.47) in the two groups, and the groups were comparable in terms of the position of the fissure as depicted in figure (Figure 3).

 For group A, the median pain score at the first follow-up (at 72 hpurs) was 8 (7-10) on VAS. On subsequent follow-ups, at the end of the 1^st^ week and at the end of the 6^th^ week, the median pain score was 8 (6-9), 5 (0-7) and 2 (0-5), respectively. For group B, the median pain score at the 1^st^ follow-up was 8 (6-10), at the end of the 1^st^ week was 6 (4-9), at the 3^rd^ week was 4 (0-6), and at the end of the 6^th^ week was 0 (0-7). It was observed that there was a statistically significant difference (*P* < 0.05) in terms of pain relief at the end of 1^st^ week between the two groups.

 However, at the end of the 3^rd^ and 6^th^ weeks, there was no statistically significant difference (*P* > 0.05) in terms of pain relief in both groups, as shown in Table 3.

**Table 3:** Pain relief after treatment

**Time of follow-up**	**Group A**	**Group B**	***P*** ** value**
After 72 hrs	8 (7-10)	8 (6-10)	0.1556
At the end of 1^st^ week	8 (6-9)	6 (4-9)	0.00018
At the end of 3^rd^ week	5 (0-7)	4 (0-6)	0.242
At the end of 6^th^ week	2 (0-5)	0 (0-7)	0.4532

 Healing of anal fissure is significantly higher in patients in group B compared with group A at the end of the 6^th^ week (*P* < 0.05). However, the difference is not significant at the end of the 3^rd^ week (Table 4). Patients with non-healing fissures at the end of 6 weeks in both groups i.e., 15 out of 25 in group A and 5 out of 25 in group B, were advised alternative method in the form of surgical treatment.

**Table 4:** Fissure healing

**Time of follow-up**	**Group A**	**Group B**	***P*** ** value**
At the end of the 3^rd^ week	4 (16%)	6 (24%)	0.674
At the end of the 6^th^ week	10 (40%)	20 (80%)	0.0152

**Figure 4:**
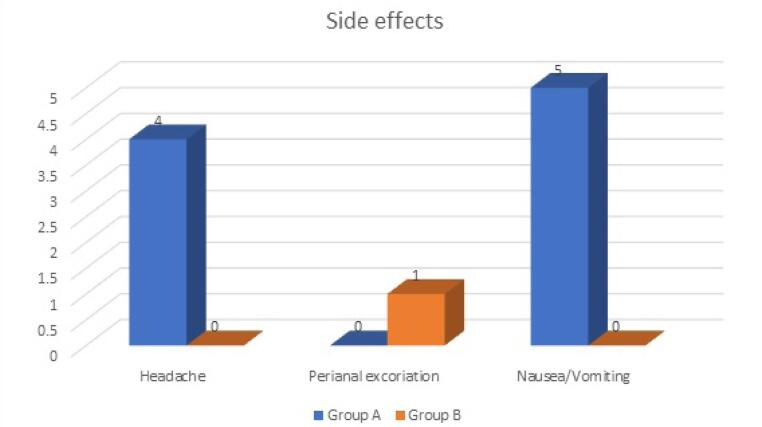
Side effects of treatment

 Side effects were seen in a total of 10 patients, 9 in group A and 1 in group B (Figure 4). Group A reported headaches in four patients and nausea/vomiting in five patients, while group B reported one patient excoriation. The difference was not statistically significant (*P* = 0.054).

 Recurrence was observed in a total of four patients, two patients in each group at the end of mandatory 3 months follow-up. This difference was not statistically significant (*P* > 0.05) between the two groups.

## Discussion

 Anal fissure/ fissure-in-ano is a linear defect or laceration in the anodermal region situated between Dentate or Pectinate line and the anal verge. It is a common anorectal condition that usually presents as severe anal pain while defecating.

 The mean age of patients in group A was 32.00 ± 10.67 years, and in group B was 30.64 ± 9.53 years. These data are consistent with the studies done by Chaudhary et al^9^ and Golfam et al.^10^ However, Pfenninger and colleagues reported occurrence of anal fissures was common in the age group of 15-40 years.^2^ The present study is comparable with previous literature. The study conducted by FitzDowse and colleagues^11^ and Al-Thoubaity^12^ reported the mean age of 44 years & 45 years, respectively.

 The incidence of anal fissures is around 1 in 350 adults, with no definite sex preponderance. In our study, 62% (n = 31) of patients were male, with group A and group B having 64% (n = 16) and 60% (n = 15) male patients, respectively. The difference was insignificant. Similar results were shown by Patel et al^13^ and Golfam et al^10^ who reported insignificant female preponderance. However, a study conducted by Al-Thoubaity^12^ had 691 patients, and all were females, while FitzDowse et al^11^ showed male predominance. Salati reported that middle-aged and younger patients were more commonly affected with equal frequency in men and women.^14^

 Pain is seen in all patients in the present study. 76% of patients in group A and 80% of patients in group B primarily presented with bleeding during defecation. The latter was associated with constipation in 80% and 76% of patients in group A and group B, respectively. The study is comparable to FitzDowse and colleagues, who reported pain was seen in 70% of their patients followed by rectal bleeding (55%) and pruritis (35%).^11^ Similar data were shown by Al-Thoubaity,who reported pain, bleeding per rectum, and pruritis as common symptoms.^12^ The pain in anal fissures recurs with every bowel movement, which makes patients afraid or unwilling to have a bowel movement, which leads to a vicious cycle of worsening constipation, harder stools, and an increase in anal pain. Approximately 70% of patients note a small amount of bright red blood on the toilet paper or stool.^14^

 The most common site affected is midline posteriorly, but anterior midline fissure is not infrequent, especially in women. In this study, 84% of patients in group A and 76% of patients in group B had posterior midline fissures. Similar findings were observed by Rana and colleagues., which showed posterior fissure as more common than anterior.^15^ As per Salati, in both sexes, midline posterior fissures are seen in 90% of cases. Anterior fissures are common in women, with an incidence of 10%-25%, while in men it is 1%-8%.^14^

 The differences in the decrease of median pain score among the two groups were not statistically significant except at the end of 72 hours of treatment, where pain relief was significant in group B. Jonas and colleagues, in their study of 50 patients, showed no significant differences in reduction of mean pain score between the groups who were managed with oral diltiazem and ointment diltiazem locally for a duration of 8 weeks.^16^ However, Tsunoda and colleagues reported a significant reduction of symptoms with the use of topical diltiazem, which improved the quality of life.^17^

 The difference between the two groups in relation to the healing of fissures was not significant statistically at the end of the 3^rd^ week, but at the end of the 6^th^ week, the difference was statistically significant among the two groups. The success rate was 80% in group B and 40% in group A. The current study is comparable to Jawaid and colleagues,^18^ which showed 77% healing, and Khan and co-workers,^19^ which showed a success rate of 80.4% with topical diltiazem. Kocher et al^20^ showed 82% success rate with topical diltiazem. However, Jonas and colleagues showed no significant difference between the use of oral diltiazem and topical diltiazem (*P* = 0.09).^16^

 Side effects of oral diltiazem were seen in nine patients in group A in the form of headache and nausea/vomiting. Jonas and colleagues reported similar findings where eight patients had side effects in the form of headache, nausea/vomiting, and rashes in the oral diltiazem group, while no side effect was seen in the local diltiazem group.^16^ Salati reported the occurrence of pruritis 10% of cases in topical application.^14^ Kocher et al^20^ and Shrivastva et al^21^ demonstrated nil systemic side effects with topical diltiazem treatment, making it a safer option for the treatment of fissures.

 In the present study, the recurrence was observed in a total of four patients, two in each group at 3 months of follow-up. The recurrence rate in group A was 20%, and in group B was 10%. Jonas et al. reported a lower recurrence rate i.e.,11.11% in the oral group and 5.88% in the local group.^16^ Shrivastva and colleagues reported a recurrence rate of 12.5 % in patients who were treated with ointment diltiazem, which was comparable to the current study.^21^ Similarly, Suevarna and colleagues showed a recurrence rate of 9.2% in patients who were using ointment diltiazem.^22^ However, a longer follow-up period may be required to see a recurrence in anal fissures. Another limitation of the study is the small sample size, and better results can be obtained with larger groups.

## Conclusion

 In conclusion, topical diltiazem is more effective in reducing symptoms of anal fissure compared with oral diltiazem. Topical diltiazem has a high success rate and is significantly effective in treating anal fissures. Although not significant in the current study, the recurrence rate and side effects are higher in oral therapy than the local application of diltiazem, making topical drugs a better option than oral therapy.
